# Cystic Fibrosis Screening Efficacy and Seasonal Variation in California: 15-Year Comparison of IRT Cutoffs Versus Daily Percentile for First-Tier Testing

**DOI:** 10.3390/ijns10040076

**Published:** 2024-11-22

**Authors:** Stanley Sciortino, Steve Graham, Tracey Bishop, Jamie Matteson, Sarah Carter, Cindy H. Wu, Rajesh Sharma

**Affiliations:** Genetic Disease Screening Program, California Department of Public Health, Richmond, CA 94804, USA

**Keywords:** newborn screening, IRT, DNA, CF, cystic fibrosis, fixed cutoff, floating cutoff, false negative

## Abstract

The California Genetic Disease Screening Program (GDSP) employs a fixed immunoreactive trypsinogen (IRT) cutoff followed by molecular testing to screen newborns for cystic fibrosis (CF). The cutoffs approximate a 1.6% yearly IRT screen-positive rate; however, seasonal variation in IRT population means has led us to develop a model to establish fixed IRT cutoffs that anticipate seasonal variation and minimize missed cases below cutoff. We utilized an ARIMA model to fit monthly IRT screen-positive percentiles and estimated regular seasonal expectations. We established a retrospective cohort followed for at least 1.5 years to capture missed false-negative CF cases. We compared missed CF cases identified by seasonal cutoffs vs. floating cutoffs. GDSP screened 7,410,003 newborns, from July 2007 to December 2022, and missed 36 CF cases below the fixed cutoff; five of the 36 were within 3 ng/mL below the cutoff. There was a regular, seasonal cycle that varied from 1.4% in summer to 1.8% in winter. We would have missed 59 CF cases using a 1.6% daily floating cutoff. California would need to use a 4% daily floating cutoff to improve our current detection rate, which would double the number of specimens sent for costly molecular analysis.

## 1. Introduction

Newborn screening (NBS) by the California Genetic Disease Screening Program (GDSP) identifies children diagnosed with cystic fibrosis (CF) and CF-related metabolic syndrome (CRMS) using a three-tier IRT–DNA–DNA testing method. When our first-tier assay of immunoreactive trypsinogen (IRT) is higher than a cutoff, we send the dried bloodspots (DBS) for analysis with a panel of disease-causing genetic mutations in the CFTR gene; if only one panel mutation is detected, we sequence the CFTR gene. The IRT cutoff is an important gateway to further testing for CF.

Our fixed IRT cutoff has changed periodically to anticipate a nominal population-based [1] yearly IRT screen-positive rate after a new reagent kit has been introduced. Observed seasonal variation in our population IRT percentiles led us to develop a seasonal model to help set initial kit-based cutoffs to run throughout the duration of each kit, which can exceed 6 months. Upon introduction of a new IRT reagent kit, we adjust our IRT cutoff to approximate a 1.6% yearly IRT screen-positive rate, to avoid unnecessary genetic testing.

It is known that IRT levels in newborns differ by race, gestational age, and gestational weight. [2,3] IRT population means can also change by season and can differ between laboratory reagent kits [3,4,5,6]. Some state screening programs establish cutoffs based on floating daily percentiles; however, those programs call out a much higher percentage (4–5% vs. 1.6%) of the newborn population for molecular testing than does California [3,6,7]. We cannot predict the diverse mix of races and birth conditions among the newborns screened in California laboratories on any given day, but we can leverage statewide population data to take regular seasonal variability into account, to set fixed cutoffs, and still identify CF cases efficiently. Seasonal variation must be monitored to maintain an effective fixed cutoff, whereas variation is automatically built into a floating cutoff. We wanted to evaluate the trade-offs for each method. There is another approach, which is a repeat IRT after a positive IRT screening test followed by molecular testing (IRT-IRT-DNA) [8,9]. IRT–IRT–DNA–DNA shows promise in lowering false-positive IRT results, but we do not request second specimens routinely in California, with some exceptions [10,11], and cannot emulate the method.

We reevaluated our fixed cutoffs in the summer of 2017 after we missed two CF cases close to the IRT cutoff boundary. In May of 2017, a new reagent kit was introduced, the number of IRT positives sent for molecular testing dropped, and we missed two cases over the following two months of summer. This was a perfect storm where a new kit required a change to a lower fixed cutoff moving into the summer, when population IRT positive percentiles are the lowest of the year. There are many reasons a case of CF can be missed [12]; for our investigation, we address false-negative CF cases due to a low IRT result below the cutoff.

We provide results of our seasonal analysis of population data, which establish initial IRT cutoffs for new reagent kits that anticipate variation throughout the life of a kit. We also compare GDSP cutoffs with the alternative method of floating laboratory-based cutoffs to see which method would correctly identify more CF cases near the IRT detection boundary. It is also important to compare which method may increase or reduce the amount of costly molecular testing among newborns without CF, testing that can identify variants of unknown significance (VUS), creating distress for families of newborns thus identified.

## 2. Methods

### 2.1. Study Population

Initial IRT screening is performed by five laboratories contracted by GDSP that send results and specimens to the central GDSP Genetic Disease Laboratory. IRT values (ng/mL) are measured using the AutoDELFIA Neonatal IRT Kit (Revvity, Waltham, MA, USA) on newborn dried bloodspot (DBS) cards. Cards with IRT values that exceed our fixed cutoff are sent to the Stanford University Molecular Pathology Laboratory, which performs the California 75-mutation panel followed by Sanger sequencing of the CFTR gene to identify an additional variant when a single allele is identified with a CF-causing panel mutation [13,14,15]. California moved from a 35- to a 75-mutation panel in 2020. Once genetic testing is complete, results of screening are provided by our Screening Information System (SIS) to the ordering physician and one of our Area Service Centers (ASCs) that notify the pediatrician to arrange a referral to one of the five contracted CF Special Care Centers for clinical management, diagnosis, and treatment. The Centers work with specialists and our ASC coordinators to report a diagnosis of CF shortly after birth, including cases missed by NBS.

We established a 15-year study cohort from 16 July 2007 through 31 December 2022 and identified IRT values and cutoffs on a given day for all newborns screened by GDSP in California. All missed cases identified by July of 2024 and tested by NBS initially within the cohort window were included, providing at least 1.5 years of follow-up after the end of the cohort. We excluded cases initially tested by NBS who were born out of state.

### 2.2. Statistical Analysis

We utilized an ARIMA (autoregressive integrated moving average) model to fit monthly IRT screen-positive percentiles. Model results were used to test for seasonality and to estimate regular seasonal expectations for monthly screen-positive percentiles. The final seasonal model results were used to estimate IRT cutoffs as follows:

Monthly target percentile = Monthly seasonal % * percent positive IRT target (1.6%). Monthly IRT cutoff value = IRT value calculated at the target percentile.

To emulate a floating cutoff, we used existing data from the study period, and calculated daily IRT distributions per contract laboratory at different cutoff levels determined by the top 1.6, 2.0, 3.0, 4.0, and 5.0 percentiles of each of the contact labs distributions. For each cutoff percentile, the frequency of known missed cases that would now be IRT-positive or remain IRT-negative was calculated and summed over the labs. The frequency of new IRT-negative missed CF cases was also calculated. Cutoff levels and screening result frequencies were also calculated using the total previous seven-day IRT weekly distribution.

Multivariate logistic regression results are presented as odds ratios (OR) and 95% confidence intervals (95% CI). The statistical significance threshold was set at *p* < 0.05. We used SAS for tests of significance, analysis, and graphics Tests of significance, the analysis, and graphics were generated using [SAS/STAT] software, Version [9.4]. Copyright © [2016] SAS Institute Inc., Cary, NC, USA.

## 3. Results

**Figure 1:**
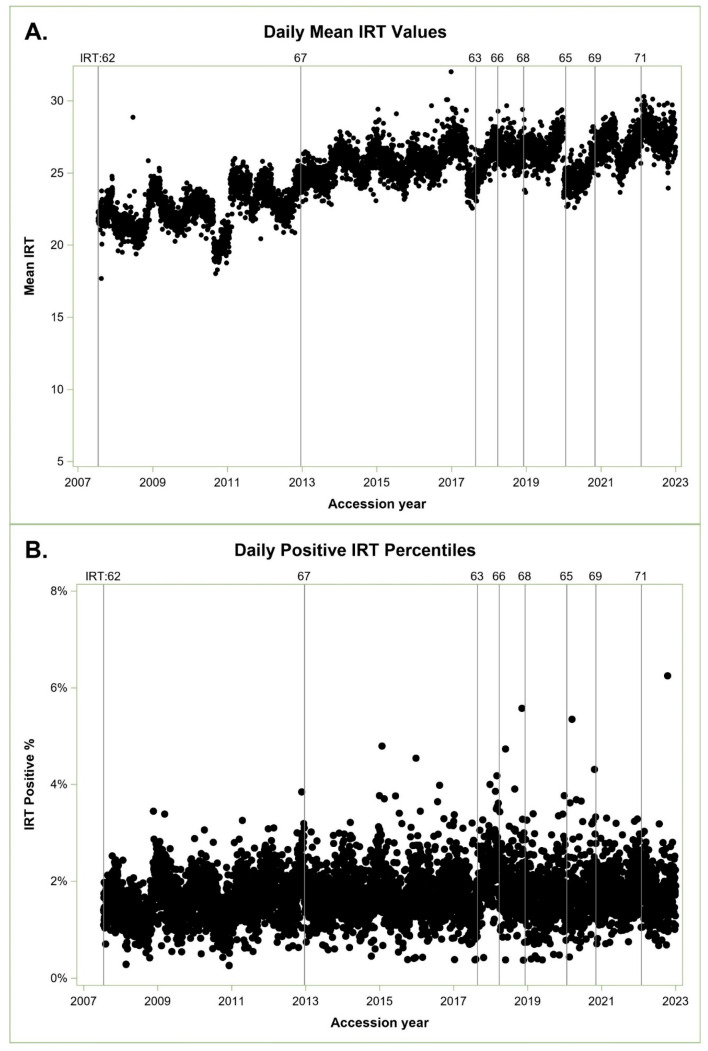
Daily longitudinal variation in IR in California. IRT values as ng/mL. (**A**) Daily mean IRT values. (**B**) Daily positive IRT percentiles. IRT index timelines indicate the IRT cutoff and implementation date.

From 16 July 2007 through 31 December 2022, we screened 7,410,003 newborns with 123,313 IRT screen-positive results for an overall IRT screen-positive rate of 1.66%. We missed 80 cases in total: 44 (55%) above the IRT cutoff and 36 (45%) below the IRT cutoff. Five of the thirty-six low-IRT cases were within 3 ng/mL of the cutoff and considered near the IRT detection boundary. Figure 1A shows statewide daily IRT means over time, and Figure 1B shows daily IRT-positive percentiles over time.

**Figure 2:**
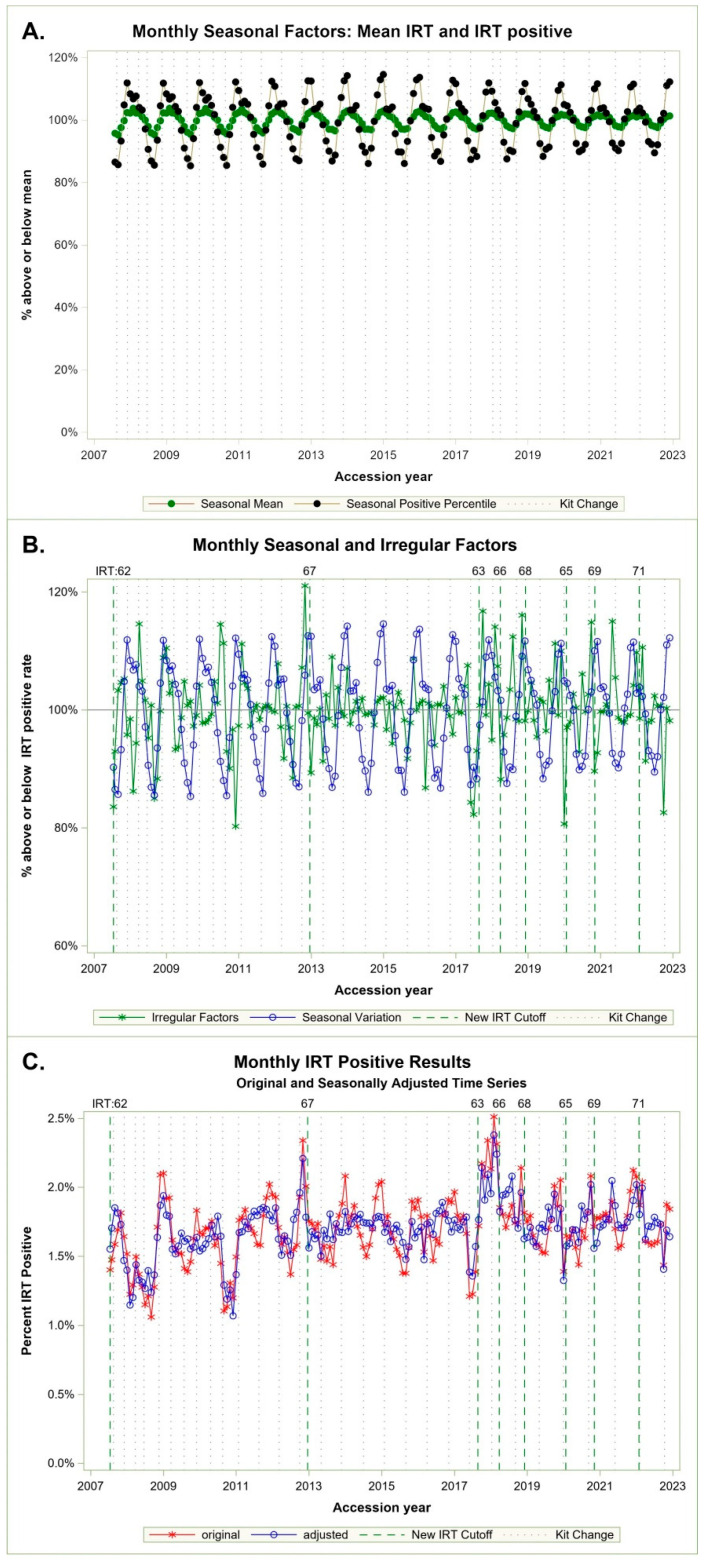
Monthly modeled longitudinal seasonal variation in IRT. (**A**) Monthly ARIMA seasonal factor estimates contrasting mean vs. IRT screen-positive percentages above or below 100%. (**B**) Monthly percent seasonal and irregular factors above or below the IRT-positive rate. (**C**) Monthly original and adjusted IRT-positive results.

Patterns of mean IRT values and IRT-positive percentiles are difficult to compare until we perform separate ARIMA models and extract monthly seasonal factors indicating a consistent cyclic monthly percentage above and below the IRT screen-positive population target, represented as 100% in Figure 2A. The ARIMA model indicated a statistically significant seasonal affect (stable seasonality F-test *p* ≤ 0.0001) and no evidence of moving seasonality for the positive percentile (moving seasonality F-test *p* ≤ 0.22). There was marginal evidence of moving seasonality for mean monthly IRT values (moving seasonality F-test *p* ≤ 0.07), which relates to the observed increase in mean IRT values over time in Figure 1A.

Figure 2A shows seasonal cycles of percentiles in black and IRT mean in green. Cycles are more pronounced for positive IRT percentiles, which vary in a range of 82% to 112%, compared with the IRT means, which vary between 95% and 104%. Both cycles are consistent and aligned, peaking in winter and dropping in summer. IRT-positive percentiles are our concern, as these cutoffs determine which specimens are sent for molecular analysis.

Figure 2B shows both the seasonal and irregular monthly factor for the positive percentiles. Here, the larger irregular factors appear to coincide with new kits and a subsequent change in cutoffs. Figure 2C indicates original monthly IRT-positive percent and time series adjusted by seasonal factors. The adjusted series can be interpreted as what we may have seen if NBS had made cutoff corrections each month based on the model vs. the IRT percent observed in our real data.

**Figure 3:**
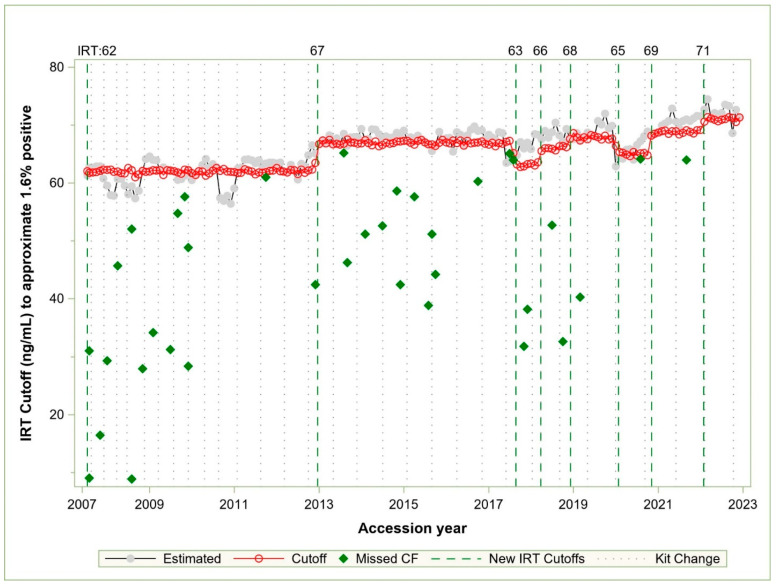
Monthly seasonal estimated and actual IRT cutoffs with missed cases. Estimated—seasonal monthly estimated IRT cutoff values to approximate 1.6% screen-positive rates. Cutoff—actual monthly cutoffs calculated from the population data. New IRT cutoffs—initial cutoff changes by the month of change. Kit change—month of a new reagent kit.

Figure 3 compares IRT values of each cutoff vs. IRT values estimated by the model results. Between July 2007 and 2017, the cutoff was changed once, from 62 to 67 ng/mL in late 2012. There was one missed case within 3 ng/mL of the cutoff in 2011, and there were kits in 2008 and 2010 that appeared to require a cutoff far lower than the 62 ng/mL in use at that time, though no known cases were missed. In the summer of 2017, we missed two cases of CF shortly after a reagent kit change in May. The kit change led to low IRT percentiles due to continuation of the IRT cutoff of 67 ng/mL. We subsequently made a cutoff change to 63 ng/mL in the summer based on the ARIMA model that we developed at that time. IRT reagent kits (Figure 1A and Figure 3) have shown increases in population means over time, but the changes are episodic and may not indicate a true trend, since the kits are under the control of the vendor who can alter the properties of a kit if requested. We can handle adjustments with calculation and cutoff changes swiftly for a new reagent kit rather than wait for the vendor to change paramters of a kit upon request. New reagent kits were more stable, were introduced less frequently, and were in use longer than they were prior to 2012 (dotted vertical lines in Figure 2 and Figure 3), which made adjustment at GDSP facile.

Table 1 shows the results of a multivariate logistic regression with seasonal and regional effects comparing missed and identified CF cases. There was a strong statistically significant seasonal effect (seasonal Chi-square = 10.0, *p* < 0.019), with OR of 6.50 (95% CI = 1.4, 28.96) in fall and 6.20 (95% CI = 1.41, 27.27) in summer compared with missed cases found below the IRT cutoff in the spring. Winter had wide confidence limits and was not significantly different from spring (OR = 2.01, 95% CI = 0.36, 11.07). There was no detectable overall regional effect (regional Chi-square = 0.95, *p* < 0.812) compared with the Los Angeles (LA) area counties.

To ascertain whether there was seasonal variablility in the incidence of confirmed CF cases that might interfere with the analysis, we conducted a multivariate logistic regression, and found no evidence of significant seasonal variation (seasonal Chi-square = 3.48, *p* < 0.323) in percentages of confirmed CF cases among the screened California population indicated by Supplemental Table S1. Population-based regional incidence among all confirmed CF cases showed a strong regional effect (regional Chi-square = 66.67, *p* < 0.0001) compared with Southern California, which was likely due to differences in the ancestry and heritable allele frequecies found among our diverse screened populations.

The IRT percent screen-positive targets listed in Table 2 were created by extracting monthly seasonal percentiles and multiplying by desired yearly IRT population percentile. We show three estimates using 1.6%, 1.7%, and 1.8% population percentiles. In California, percentiles are highest in December and January and lowest during the summer months of June through August.

In Table 3, we estimated daily and weekly floating cutoffs using data summed from each of the five contract labs and our known missed and confirmed CF cases. We found that a daily floating cutoff was able to identify many of the current low-IRT missed cases at 4–5% screen-positive rates and did not create new false-negative, missed cases. These cutoffs would require an additional 197,784 to 276,457 genetic tests to be performed, over twice as many as we currently conduct if we considered the 5% daily floating cutoff. The weekly floating estimate did not add new false-positive cases starting at 3% and captured half of the current IRT false-negative missed cases at a 5% cutoff percentile.

## 4. Discussion

ARIMA analysis allowed us to examine the regular seasonal pattern of IRT screen-positive percentiles which has been observed in other states. Logistic analysis confirmed that low-IRT missed cases were more likely to occur during the summer and fall compared with spring months, even after we included region in the multivariate model. This remained true even after we removed the five results that were within 3 ug/mL of the IRT cutoff (not shown). Summer and fall comprise the hottest months, when specimens are transferred at room temperature and IRT enzyme in specimens can degrade. We used the analysis to define stable cutoffs based on the first two weeks of kit introduction. If we had used the adjustment factor derived from this model in 2017, we would not have missed CF cases near the IRT detection boundary; instead, we missed two cases in practice due to a delay in making a cutoff change (Figure 2). After many years without a cutoff change, the team was caught off guard when a kit changed dramatically in 2017. We have since monitored IRT screen-positive levels monthly with an emphasis on summer and fall.

We summarized the output of the ARIMA model in Table 1 with monthly percentile targets, which can be used to determine the percentile we want to achieve when we have a new kit. We estimated the IRT value that falls within the population percentile for the new kit. The new cutoffs we estimated have been sufficient to set once throughout the life of a kit. The table can also be used to monitor and change cutoffs monthly, but we have not needed to change cutoffs once they have been set. The most important set points are in the summer, and we have observed that monthly percentiles that drop below 1.4% to 1.5% raise the potential of a missed case close to the cutoff boundary. However, the bulk of missed CF cases due to low IRT values are well below the cutoffs in summer and fall and difficult to detect using IRT alone.

To capture low-IRT CF cases that evade detection, we have had discussions with other NBS programs in the United States about whether to collect risk factors for CF—such as meconium ileus at birth or maternal CFTR modulator drug exposure during pregnancy—on the bloodspot test request form to initiate molecular testing for CF regardless of IRT result when a risk factor is noted. However, these and other known risk factors for CF should already trigger a clinical investigation regardless of an NBS result, and we educate clinicians not to rely solely on newborn screening for an infant at heightened risk for CF or other disorders [16]. The objective of NBS is to screen the entire newborn population without prior knowledge of an individual infant’s known risk for disease.

We could augment IRT first-tier followed by second-tier pancreatitis-associated protein (PAP) testing on the initial bloodspot. If PAP has a higher specificity for CF than IRT, we could maintain sensitivity using fixed cutoffs as discussed and improve specificity with PAP [17]. PAP may also be resistant to seasonal variation [5]. If PAP second-tier demonstrates good performance in the large and diverse California populations and does not delay molecular testing significantly, such a second-tier may be beneficial. We may expand the expected yearly percentile of our fixed cutoff and still send fewer specimens for molecular testing, return fewer CRMS results, and correctly identify the few infants with CF who are at the edge of the IRT cutoff boundary. Though PAP second-tier testing may not reduce the CF cases of high IRT missed during molecular testing, the proposal is worth further investigation [17].

When we commenced CF screening in 2007, we determined a cutoff of 62 ng/mL based on population projections and historical test data, which led us to target a yearly percentile of 1.6%. The program has maintained a positive percentile between 1.6% and 1.7% yearly with rare changes in cutoff until 2017 when we dropped the cutoff from 67 to 63 ng/mL. We have been monitoring percentiles since then, and results of our analysis suggest that we can move our percentile to 1.7% yearly, assuring that the lowest monthly percentile is 1.5% during the summer months.

Table 3 suggests that daily variability in the screening population and in IRT results was great enough to lower screening efficacy for CF, and a weekly floating cutoff was more effective. If California went to a laboratory-based daily percentile, we would need to set a 4–5% daily floating cutoff to lower missed cases at a cost of sending 152–212% more specimens for molecular testing. If our five contract laboratories were able to calculate a floating cutoff derived weekly, we could use a lower 3–4% cutoff level, but we would still increase our molecular testing by 82–142% respectively (Table 3). The daily variablility would be exacerbated if floating cutoffs were estimated at the instrument level rather than the laboratory; the smaller the unit of analysis, the greater the variance and higher the daily floating percentile cutoff required. This analysis, though limited to California, can help explain why states that use floating cutoffs may require a 4–5% IRT screen-positive rate in contrast to our lower 1.6% rate.

### Limitations

This study’s main limitation is the incomplete identification of missed cases. There may be a long lead time before a missed CF case is identified. Missed cases may be identified in other states or counties and never make it into our surveillance system. Missed cases in California may never be entered into our surveillance system, even though we maintain good relationships with our CF Special Care Centers who report such cases to us. Incomplete case ascertainment may hamper the examination of floating cutoffs we presented using California data. If we had doubled the number of molecular tests our program conducted at lower IRT levels, we may have captured more confirmed CF cases. We suspect that that number would be small given the episodic nature of low-IRT false-positive results, but we do not know for certain.

Results from California may not be generalizable. California has a diverse population which can increase variability in IRT results based on the daily mix of births among people of differing genetic ancestry.

The analysis may not apply directly to other states or countries due to differing temperature gradients and seasonal extremes. However, the key minimum set point for any program is during the months when environmental temperatures are highest. The other maximum IRT percentiles can then be set based on the desired yearly population percentiles.

## 5. Conclusions

Regular seasonal variation in IRT screen-positive rates can be leveraged to establish initial fixed cutoffs for a new reagent kit that can be monitored throughout the year. Daily and weekly population variability is large enough that IRT percentiles may reduce screening efficacy compared with a fixed cutoff as long as the fixed cutoff is monitored for monthly IRT-positive percentiles, especially during the summer and fall. A population-based model and fixed cutoffs have been more efficacious, reduced missed cases, and minimized molecular testing for California. States and countries with small birth populations compared to California may do well with fixed cutoffs rather than floating due to the variablilty inherent in small numbers. We try to reduce the burden of stress and uncertainty for families with newborns with identified VUSs and with CRMS, a “watchful waiting” symptomless state at heightened risk for CF, whom we may identify at the low end of the screening threshold.

## Figures and Tables

**Table 1:** Seasonal and regional effects on detection of missed CF cases below the IRT cutoff among all CF cases identified in California: multivariate logistic regression.

Effect		All CF Cases ^a^	Missed Low-IRT Cases ^b^	% Missed	Odds Ratio	95% Confidence Limits		Effect Wald Chi-Square ^c^	*p*-Value
Season	Fall	247	14	5.7%	6.50	(1.46,29.0)		10.00	0.019
	Summer	289	16	5.5%	6.20	(1.41,27.3)			
	Winter	219	4	1.8%	2.01	(0.36,11.1)			
	Spring	215	2	0.9%	1.00	reference			
Region ^d^	Bay Area	154	5	3.2%	1.05	(0.38,2.94)		0.95	0.812
	Farm Belt	278	13	4.7%	1.44	(0.68,3.06)			
	Northern Mountain	55	2	3.6%	1.18	(0.26,5.32)			
	Southern California	483	16	3.3%	1.00	reference			
	Total	970	36	3.7%					

^a^ All CF cases born in California identified by NBS, including missed cases. ^b^ Missed CF cases below the IRT cutoff. ^c^ Seasonal- and regional-effect-level multivariate Chi-square. ^d^ Counties included in each region. Bay Area: Alameda, Contra Costa, Marin, Napa, San Francisco, San Mateo, Santa Clara, Santa Cruz, Solano, and Sonoma. Farm Belt: Colusa, El Dorado, Fresno, Imperial, Kern, Kings, Madera, Merced, Monterey, Placer, Sacramento, San Benito, San Joaquin, San Luis Obispo, Stanislaus, Sutter, Tulare, Yolo, and Yuba. Southern California: Los Angeles, Orange, Riverside, San Bernardino, San Diego, Santa Barbara, and Ventura. North Mountain: Alpine, Amador, Butte, Calaveras, Del Norte, Glenn, Humboldt, Inyo, Lake, Lassen, Mariposa, Mendocino, Modoc, Mono, Nevada, Plumas, Shasta, Sierra, Siskiyou, Tehama, Trinity, and Tuolumne.

**Table 2:** IRT monthly target percentiles.

		Seasonal Multiplier of Yearly Percentile	IRT Screen-Positive Percentiles (Yearly Target Percentiles) ^a^		
**Yearly Target Percentiles:**		**100.0%**	**1.60%**	**1.70%**	**1.80%**
Monthly targets	Jan	110.0%	1.8%	1.9%	2.0%
	Feb	104.5%	1.7%	1.8%	1.9%
	Mar	104.4%	1.7%	1.8%	1.9%
	Apr	103.0%	1.6%	1.8%	1.9%
	May	96.3%	1.5%	1.6%	1.7%
	Jun	91.9%	1.5%	1.6%	1.7%
	Jul	90.7%	1.5%	1.5%	1.6%
	Aug	89.0%	1.4%	1.5%	1.6%
	Sep	93.0%	1.5%	1.6%	1.7%
	Oct	99.2%	1.6%	1.7%	1.8%
	Nov	107.3%	1.7%	1.8%	1.9%
	Dec	112.2%	1.8%	1.9%	2.0%

^a^ Yearly targets (top row bolded percentiles) are used to calculate the monthly target percentiles in each column.

**Table 3:** Daily and weekly floating cutoff estimations.

Period of Estimation ^a^	Cutoff Percentile Estimated ^b^	IRT False Negative (Remaining Currently Missed) ^c^	New True Positive (Among Currently Missed) ^d^	New False Negative ^e^	Total Missed Cases ^f^	Estimated Additional Specimens for Genotyping (% Greater than Baseline) ^g^	
Daily	1.6%	34	2	24	58	8413	(7%)
	2%	31	5	13	44	38,366	(31%)
	3%	27	9	3	30	112,300	(91%)
	4%	21	15	0	21	186,195	(151%)
	5%	17	19	0	12	260,407	(211%)
Weekly	1.6%	35	1	8	43	−3173	−(3%)
	2%	33	3	1	34	26,462	(21%)
	3%	25	11	0	25	100,629	(82%)
	4%	20	16	0	20	174,707	(142%)
	5%	18	18	0	18	248,910	(202%)
	Baseline	36	0	0	36	123,313	(100%)

^a^ Period of estimation based on daily or weekly laboratory data. ^b^ Cutoff percentile estimated based on either daily or weekly laboratory-specific data. ^c^ IRT false-negative missed cases among 36 missed currently. ^d^ New true-positive results identified among the 36 missed currently. ^e^ New false-negative cases previously identified as true positive. ^f^ Total missed cases calculated as sum of new true- and false-negative cases. ^g^ Estimated additional specimens sent for genotyping due to a floating cutoff. Percentage based on additional divided by current IRT screen-positive cases among 123,313 baseline specimens.

## Data Availability

Participant data cannot be made available due to legal and ethical requirements restricting access to individual-level data from the California Newborn Screening Program.

## Supplementary Materials

The following supporting information can be downloaded at: https://www.mdpi.com/article/10.3390/ijns10040076/s1, Table S1: Seasonal and regional effects on incidence of CF cases identified in California: Multivariate logistic regression.

## Abbreviations

ARIMA	Autoregressive integrated moving average
CF	Cystic fibrosis
CFTR	Cystic fibrosis transmembrane conductance regulator gene
CRMS	CFTR-related metabolic syndrome
GDSP	Genetic Disease Screening Program
IRT	Immunoreactive trypsinogen
NBS	Newborn screening
PAP	Pancreatitis-associated protein
